# Costs and effects of telerehabilitation in neurological and cardiological diseases: A systematic review

**DOI:** 10.3389/fmed.2022.832229

**Published:** 2022-11-29

**Authors:** Rocio Del Pino, Maria Díez-Cirarda, Iker Ustarroz-Aguirre, Susana Gonzalez-Larragan, Massimo Caprino, Stefan Busnatu, Kai Gand, Hannes Schlieter, Iñigo Gabilondo, Juan Carlos Gómez-Esteban

**Affiliations:** ^1^Neurodegenerative Diseases Group, Biocruces Bizkaia Health Research Institute, Barakaldo, Spain; ^2^Economic Evaluation Department, Cruces University Hospital, Barakaldo, Spain; ^3^Department of Health Science Library, Cruces University Hospital, Barakaldo, Spain; ^4^Department of Neurorehabilitation Sciences, Casa di Cura del Policlinico Spa, Milano, Italy; ^5^Faculty of Medicine, Carol Davila University of Medicine and Pharmacy, Bucharest, Romania; ^6^Technische Universität Dresden, Faculty of Business and Economics, Research Group Digital Health, Dresden, Germany; ^7^Ikerbasque, The Basque Foundation for Science, Bilbao, Spain; ^8^Department of Neurology, Cruces University Hospital, Barakaldo, Spain; ^9^Department of Neuroscience, University of the Basque Country (Universidad del Pais Vasco/Euskal Herriko Unibertsitatea), Leioa, Spain

**Keywords:** cost-effectiveness, telerehabilitation, cardiological diseases, neurological disease, systematic review

## Abstract

**Introduction:**

Telerehabilitation in neurological and cardiological diseases is an alternative rehabilitation that improves the quality of life and health conditions of patients and enhances the accessibility to health care. However, despite the reported benefits of telerehabilitation, it is necessary to study its impact on the healthcare system.

**Methods:**

The systematic review aims to investigate the costs and results of telerehabilitation in neurological and cardiological diseases. MEDLINE and EMBASE databases were searched from 2005 to 2021, for studies that assess the costs and results of telerehabilitation compared to traditional rehabilitation (center-based programs) in neurological and cardiological diseases. A narrative synthesis of results was carried out.

**Results:**

A total of 8 studies (865 participants) of 430 records were included. Three studies were related to the costs and results of telerehabilitation in neurological diseases (specifically in stroke). In total, five studies assessed telerehabilitation in cardiological diseases (chronic heart failure, coronary heart disease, acute coronary syndrome, and cardiovascular diseases). The duration of the telerehabilitation ranged from 6 to 48 weeks. The studies included cost-analysis, cost-benefit, cost-effectiveness, or cost-utility. In total, four studies found significant cost/savings per person between $565.66 and $2,352.00 (*p* < 0.05). In contrast, most studies found differences in costs and clinical effects between the telerehabilitation performed and the rehabilitation performed at the clinic. Just one study found quality-adjusted life years (QALY) significant differences between groups [Incremental cost-effectiveness ratio (ICER) per QALY ($−21,666.41/QALY).

**Discussion:**

Telerehabilitation is an excellent alternative to traditional center rehabilitation, which increases the accessibility to rehabilitation to more people, either due to the geographical situation of the patients or the limitations of the health systems. Telerehabilitation seems to be as clinical and cost-effective as traditional rehabilitation, even if, generally, telerehabilitation is less costly. More research is needed to evaluate health-related quality of life and cost-effectiveness in other neurological diseases.

**Systematic review registration:**

[https://figshare.com/articles/journal_ contribution/Review_Protocol_Costs_and_effects_of_Telerehabilitation_in_ Neurological_and_Cardiological_Diseases_A_Systematic_Review/19619838], identifier [19619838].

## Introduction

Telerehabilitation can be defined as “the delivery of rehabilitation services at a distance utilizing electronic information and communication technologies” (1). The use of technology allows communication between clinicians and patients. It can be used to supply continuity of care at home, primarily for chronic disease patients, after a comprehensive assessment is performed by the clinician/professional. Telerehabilitation guidelines have been described to provide discipline-specific standards and requirements to rehabilitation professionals (2). Among its benefits, it can provide treatment access to rural areas and an earlier rehabilitation start (3).

Rehabilitation is prescribed to enhance the patient’s quality of life and reduce the impact of a health condition, focusing on the particular aspects based on the patient’s needs, goals, and preferences. For acute and chronic patients, with neurological and cardiological diseases, early access to rehabilitation is crucial for symptom recovery and long-term continuity of care in many cases (4, 5). Specifically, cardiac rehabilitation has demonstrated the efficacy on cardiological diseases in improving quality of life and reducing mortality (6). Moreover, stroke rehabilitation was also reported beneficial for the patients (7).

In most countries, rehabilitation is not integrated as a standard of care in the public health system, and this situation worsens in low- and middle-income countries (8). The World Health Organization reported that several unmet needs exist regarding access to rehabilitation due to the lack of funding and policies at a national level, the lack of available rehabilitation services outside urban areas, and the high out-of-pocket expenses (8). Moreover, in the past years, the prevalence of diseases with health complications has been increasing, and consequently, there has been an increment in the demand for rehabilitation services (9). In light of this, integrating rehabilitation as an essential service in the health system is included as one of Europe’s priorities for health system strengthening (10).

Telerehabilitation may overcome the lack of accessibility to rehabilitation programs for most patients in need, mostly in remote or rural areas without medical facilities. Moreover, the COVID-19 pandemic has reflected the significant contribution of telerehabilitation as a means of treatment accessibility, not only in isolated areas but also when physical attendance is impossible (11). Telerehabilitation has become a valuable solution for patient health care in this scenario.

Despite the reported benefits of telerehabilitation (6, 7), there is a need to study the impact of the implementation of telerehabilitation in the healthcare system.

In total, four different modalities have been found in the literature when addressing telerehabilitation’s costs or effects: cost-effectiveness, cost-utility, cost-benefit, and cost-analysis.

Cost-effectiveness analysis is a systematic method of comparing two or more interventions by measuring their costs and consequences (health outcomes), where the consequences of each are measured in the same standard units related to the clinical objective of the interventions (e.g., life-years gained or hospital stays) (12).

Cost-utility analysis is similar to the cost-effectiveness analysis. However, instead of measuring its incremental effects in units related to its objective or commonly used in the clinic (i.e., blood pressure or detected cases), effectiveness is measured in quality-adjusted life-years (QALYs). This encompasses life expectancy and quality of life. This measure of effectiveness makes it possible to compare different programs or interventions, as it is not specific to a specific intervention (13).

Cost-benefit analysis measures and compares a healthcare intervention’s net costs with the benefits arising from the intervention, where both net costs and benefits are expressed in monetary units (14).

Finally, cost minimization analysis or cost analysis can be performed when, regardless of the units in which health outcomes are measured, they are the same in the different options compared (15). Therefore, cost-analysis only compares the costs.

Therefore, the present systematic review aimed to investigate the costs and effects (including cost-effectiveness, cost-utility, cost-benefit, and cost minimization) of telerehabilitation in neurological and cardiological diseases.

## Methods

This review was performed following the Preferred Reporting Items for Systematic Reviews and Meta-Analyses checklist (PRISMA) (refer to Supplementary material 1). This study is part of the European Virtual Coaching for Rehabilitation in Elderly (vCare) project (no. 769807), which is focused on telerehabilitation in four different pathologies: stroke and Parkinson’s disease as neurological diseases, and heart failure and ischemic heart as cardiological diseases.

### Study selection and procedures

**FIGURE 1:**
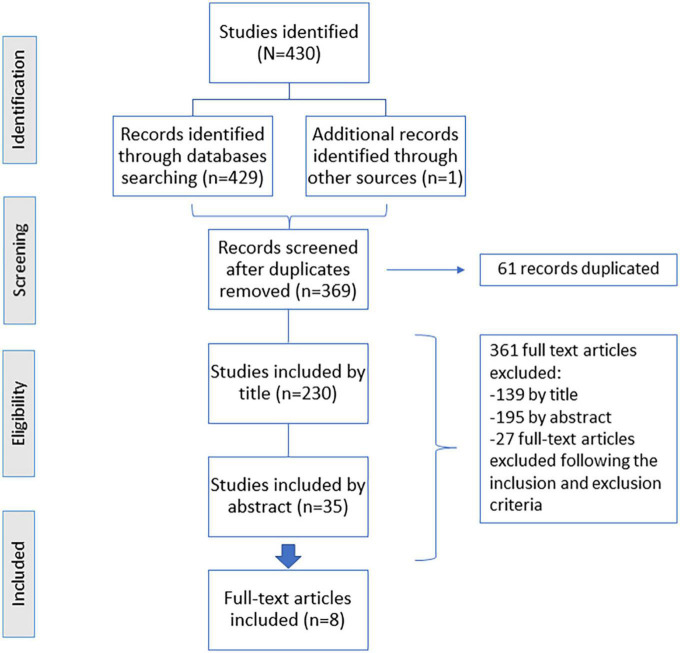
Flow diagram showing the process of study selection.

The study included all empirical studies that met the following inclusion criteria: (1) The study reported telerehabilitation vs. traditional center-based rehabilitation on neurological or cardiological diseases; (2) studies published from 2005 to 2021; (3) studies published in a peer-reviewed English or Spanish language journal; and (4) studies focused on the costs and effects of rehabilitation and virtual rehabilitation. No limits were set on the ages of participants. The exclusion criteria were as follows: (1) duplicated studies; (2) abstracts or conference papers; (3) study protocols or systematic reviews; (4) studies with inpatient participants; and (5) studies with participants with other diseases different from neurological or cardiological diseases. The protocol for this systematic review can be found in the public repository “FigShare.” The search was performed in MEDLINE and EMBASE databases in cooperation with a trained librarian and was finished in January 2021. Topics in Supplementary Table 1 detail the keywords used for each search, and the specific keyword strategy is explained in the Supplementary Table 2. In total, seven searches were performed in MEDLINE, and four were performed in EMBASE using specific keywords. The used keywords are organized by the topics related to cost, telerehabilitation, cardiological rehabilitation, and neurological rehabilitation (refer to Supplementary Table 1). In total, three experienced reviewers independently screened the search results using the inclusion and exclusion criteria explained above, following the subsequent steps: title and abstract screening, followed by full-text screening. When finalizing each step, decisions were shared among the three experienced reviewers. When the judgments of any of the reviewers were not similar, the discrepancies were explained, and a joint decision was taken. The bibliographic databases yielded 430 references in total (Figure 1).

### Data extraction and outcome

The three reviewers used a preformatted Excel sheet to extract the data for the prespecified relevant data and outcomes for each included article: (1) neurological or cardiological disease; (2) total sample size; (3) percentages of males; (4) sample size included in the telerehabilitation or control group; (5) age of each group; (6) objective of the study; (7) methods (randomization, outcome measurement, type of cost analysis, time of rehabilitation, and type of rehabilitation); and (8) results.

Risk of bias assessment was performed using the Cochrane’s risk of bias tool (16), which deals with the following sources of bias: (1) selection bias (random sequence generation and allocation concealment); (2) performance bias (blinding of participants and outcome assessors); (3) attrition bias (incomplete outcome data); (4) reporting bias (selective reporting); and (5) other sources of bias.

The reporting quality of the economic evaluation studies was assessed with the updated Consolidated Health Economic Evaluation Reporting Standards (CHEERS 2022) checklist (17) and performed independently by three researchers having taken a joint decision. Furthermore, overall quality rating of eligible studies was scored as “excellent,” “good,” “moderate,” or “‘low” quality when a study fulfilled 100%, 76–<100%, >51–75%, or 50% of the criteria, respectively.

## Results

The literature search retrieved 430 records, which were reduced to 369 after removing the duplicated ones (Figure 1). A meticulous title and abstract screening were done. After the title screening, 230 were included by title criteria inclusion, but after analyzing the abstract, 195 manuscripts were excluded, and 35 studies were included by the abstract (15 studies were related to neurological diseases, 15 studies were related to cardiological diseases, and 5 studies were classified as “others”), being all of them trial-based. Finally, eight full texts were eligible for our systematic review (Tables 1, 2).

**TABLE 1:** Summary of study characteristics.

		Approach		Sample size		Age (years) M ± SD			Methods		
				
Study	Disease		Telerehab group		Control group	Telerehab group	Control group	Objective	Type of study	Outcome measurement	Type of analysis
**Neurological diseases**											
Housley et al. (18)	Stroke	Trial-based	20		–	67.0 ± 11.4	–	–Examine the efficacy of home-based, tele robotic-assisted device to: –improve functional ability –reduce depression –increase access to –monitor participant utilization of cost-efficient rehabilitation when compared to the cost of clinic-based therapy.	Single group and projected control group	–ARAT —10MWT —6MWT –FIM –CES-D. –Costs	Cost-analysis
Lloréns et al. (19)	Stroke	Trial-based	15		15	55.5 ± 9.6	55.6 ± 7.2	–Evaluate the clinical effectiveness of a virtual reality-based telerehab program in the balance recovery of hemiparetic individuals post-stroke in comparison to an in-clinic program. –Compare the subjective experiences. –Contrast the costs.	RCT	–Berg –Balance Scale –POMA –SUS –Intrinsic Motivation Inventory –Costs	Cost-benefit
Bendixen et al. (20)	Chronic diseases (including stroke)	Trial-based	115		115	72.4 ± 9.4	71.7 ± 9.6	–Investigate the health-related cost analyses between the telerehab program (LAMP) and standard care.	Retrospective quasi-experimental design	–Hospital Beds days of care –Clinic Visits –Emergency room visits –Nursing home care unit –cost analysis	Cost-effectiveness
**Cardiological diseases**											
Hwang et al. (21)	Chronic heart failure	Trial-based	24		29	68.0 ± 14.0	67.0 ± 11.0	–Investigate the cost-utility of a home-based telerehab program vs. traditional center-based rehab program.	RCT	–Health care costs –QALY (EQ-5D)	Cost-utility
Maddison et al. (22)	Coronary heart disease	Trial-based	82		80	61.0 ± 13.2	61.5 ± 12.2	–Compare the effects and costs of remotely monitored exercise-based cardiac telerehabilitation (REMOTE-CR) with center-based program (CBexCR) in adults with coronary heart disease.	RCT	– VO_2_ peak –exercise adherence –motivation –quality of life –hospital service –medication costs –QALY (EQ-5D)	Cost-utility
Kraal et al. (23)	Acute coronary syndrome or revascularization procedure	Trial-based	45		45	60.5 ± 8.8	57.7 ± 8.7	–Examine the effect of home-based exercise training with telemonitoring guidance compared to regular center-based exercise training on physical fitness and physical activity levels	RCT	–Peak VO_2_ –VAT –PAEE –PAL –ACC –MacNew –HADS –PHQ –QALY (SF-36)	Cost-utility
Kidholm et al. (24)	Cardiovascular diseases	Trial-based	72		69	62.4 ± 12.3	62.6 ± 11.7	To assess the cost-utility of a cardiac telerehabilitation program.	RCT	–QALY (SF-36) –Costs of the intervention	Cost-utility
Frederix et al. (25)	Coronary artery disease and chronic heart failure	Trial-based	69		70	61.0 ± 9.0	61.0 ± 8.0	To evaluate the cost-utility analysis of a comprehensive cardiac telerehabilitation program.	RCT	–Health care costs –QALY (EQ-5D) – VO_2_ peak –Cardio-pulmonary exercise testing –Body mass index	Cost-utility

ACC, accelerometry; ARAT, Action Research Arm Test; CES-D, Center for Epidemiologic Studies Depression Scale; EQ-5D (EuroqOL five-dimensional); FIM, Functional Independence Measure; HADS, Hospital Anxiety and Depression Scale; LAMP, Low Activities of Daily Living Monitoring Program; NA, data not available; PAEE, physical activity energy expenditure; PAL, physical activity level; Peak VO_2_, peak oxygen consumption; PHQ, Patient Health Questionnaire; POMA, performance-oriented mobility assessment; QALY, quality-adjusted life-years; RCT, randomized controlled trial SF-36, health-related quality of life; SUS, System Usability Scale; Telerehab, telerehabilitation; VAT, ventilatory anaerobic threshold; 6MWT, 6-min walk test; 10MWT, 10-m walk test.

**TABLE 2:** Summary of telerehabilitation characteristics and study results.

		Telerehab duration and type	Results						
		
Study	Study perspective	Duration (weeks)	Type	Telerehab cost-savings/person	Significant differences	Incremental outcomes	Incremental costs	Incremental C-E Ratio	Sensitivity analysis results
**Neurological diseases**									
Housley et al. (18)	Healthcare system perspective	12	Home-based robotic rehab device Not live guided	$2,352.00	Yes	Significant Improvement in upper extremity function (30%) Clinically Significant benefits in gait speed (29%) Moderate improvement in depressive symptoms (28%) Modest improvement in distance walked (30%)	−$2,352.00	–	–
Lloréns et al. (19)	Payer’s perspective	6	Home-based telerehab vs. in-clinic rehab. Not live guided	$654.72	–	Significant improvement in both groups (in clinic and home-based telerehabilitation) but no significant differences between groups	−$655.00	–	–
Bendixen et al. (20)	Healthcare system perspective	52	Standard care + telerehab vs. standard care Not live guided	–	No	Telerehab increased clinic visits and decreased hospital and nursing home stays	–	–	–
**Cardiological diseases**									
Hwang et al. (21)	Healthcare system perspective	12	Online group-based exercise vs. traditional center-based program Live guided	$1,590.00	Yes	No differences	−$1,590.00	−$4,157 per QALY gained	
Maddison et al. (22)	Healthcare system perspective	12	Exercise-based cardiac telerehab vs. center-based program Live guided & not live guided	$2,704.05	Partially yes	VO_2_ max was comparable in both groups. REMOTE-CR was non-inferior to CBexCR. REMOTE-CR participants were less sedentary. No significant differences in hospital service utilization costs. No significant differences in QALY.	−$2,704.05	QALY gain did not differ between groups, and thus, no incremental cost effectiveness ratio was calculated.	The ICER for estimated health care costs (including estimated program costs based on full attendances and heart failure readmission costs) was -$5,408.00 per QALY gained. The ICER for all-cause health care costs (including actual program costs, and aggregated costs of all-cause emergency visits, hospital readmissions and day procedures) was -$82,536 per QALY gained.
Kraal et al. (23)	Societal perspective	12	Home-based training with telemonitoring guidance vs. center-based training Not live guided	$3,167.04	No	Although training adherence was similar between groups, satisfaction was higher in the home-based group. Physical fitness improved at discharge and at 1-year follow-up in both groups, without differences between groups. Physical activity levels did not change during the 1-year study period	−$3,167.04	–	Sensitivity analyses indicate REMOTE-CR delivery costs could be reduced to $473.54 per capita if exCR specialist time was utilized at full efficiency and wearable sensors were utilized over 5 years
Kidholm et al. (24)	Healthcare system perspective	12	Cardiac telerehab vs. Healthcare center-based rehab Not live guided	−$1,703.79	No	The number of contacts with the physiotherapist was higher among the intervention group	$1,703.79	$517,310.82 per QALY gained	The probability that it was more cost-effective varied between 97 and 75% (willingness-to-pay of €0 and $99,813.00 per quality-adjusted life-years, respectively).
Frederix et al. (25)	Healthcare system perspective	24	Internet-based + conventional center-based rehab vs. conventional center-based rehab Not live guided	$565.66	Yes	Incremental cost-effectiveness ratio of **−**$21,666.41/QALY	−$565.66	−$21,666.41 per QALY gained	The mean costs per patient in the intervention and control groups were estimated to be $5,713.3 and $4,049.41 respectively, and the mean QALY gain to be 0.089 and 0.085, whereas the mean ICER was $483.60.

The cost data were converted to USD (2022). LAMP, Low Activities of Daily Living Monitoring Program; live guided: guided and synchronized telerehabilitation monitored by clinicians; not live guided: telerehabilitation not live guided the by clinicians; QALY, quality-adjusted life-years; Telerehab, telerehabilitation; ICER, incremental cost-effectiveness ratio.

Figure 1 shows the details of the screening process. In total, three of the final studies included were related to neurological diseases, while the rest of the studies (5 out of 8) were related to cardiological diseases. These studies aimed to examine the effects and the cost of a telerehabilitation program compared to standard care. All the included studies engaged the patients in experimental and control groups, except for one study that only included patients in the experimental group and projected data for the control group (18).

Concerning the variables included (Tables 1, 2), the cost-analysis and cost-benefit studies included the costs, such as the intervention cost. The study that analyzed the cost-effectiveness used clinic visits, hospital stays, and nurse home stays. The cost-utility studies used QALY as the outcome measure of the quality of life adjusted by year, measured through the EQ-5D or SF-36 questionnaire. On the other hand, specific measures of clinical effectiveness, such as maximum aerobic capacity, body mass index, and adherence to treatment or motivation, among others, are also used to assess the effectiveness of the telerehabilitation program performed vs. the traditional rehabilitation program.

The duration of the telerehabilitation ranged from 6 to 48 weeks (Table 2), being 12 weeks the most common period to perform the telerehabilitation. The predominant type of rehabilitation was “not live guided” by the clinicians, which means that telerehabilitation was not guided by the clinicians when the patient was performing it.

### Telerehabilitation in neurological disorders

Regarding the neurological studies, which included costs and effects of telerehabilitation (Tables 1, 2), we only found studies that focused on telerehabilitation after stroke. In total, two of the studies were explicitly related to the cost and effects of telerehabilitation with stroke patients. One of the selected studies included chronically ill people and disabled elders, including patients with stroke and also diagnosed with arthritis, hypertension, and diabetes (20). The authors did not specify the sample of patients included for each diagnosis. Therefore, we used the total sample to analyze this specific study (20).

The sample size of the neurological studies included between 20 and 230 participants (56.6–95% were men). The mean age of the telerehabilitation group ranged between 55.9 ± 9.6 and 72.4 ± 9.4. Only one study performed a two-arm study with a randomized controlled trial (19). In other studies, Housley et al. (18) followed a single-group study design, and Bendixen et al. (20) carried out a retrospective quasi-experimental design.

Regarding the results (Table 2), Housley et al. (18) reported cost reductions of $2,352.00 from the telerehabilitation group compared with clinic-based therapy. Their study showed an average cost-saving of 64.97%. The costs were calculated based on the cost of equipment, device maintenance and data connection, home delivery, support and pickup, and weekly clinician follow-up and monitoring. The authors compared the costs with clinical-based outpatient therapy at a medical center. Their total cost of 3 months of telerehabilitation home-based was an average of $1,268.07 per Veteran, compared to an average of $3,619.95 per Veteran for outpatient clinic-based therapy. The primary savings were related to eliminating repeated in-person therapist costs and the absence of mileage reimbursement. Regarding clinical effectiveness, patients from telerehabilitation showed the clinical improvements related to upper extremity function, gait speed, less depressive symptoms, and improvement in distance walking. However, there was no control group to compare with.

Lloréns et al. (19) described that the estimated total cost of the balance intervention for one participant at the clinic was $1490.23, while the home-based program was $835.61. Therefore, they reported lower costs in telerehabilitation (reductions of $654.72). However, no significant differences were found in the clinical results between telerehabilitation and in-clinic rehabilitation, showing both modalities’ significant improvements in balance, gait, and mobility.

Bendixen et al. (20) detected no significant cost differences between both treatments [standard care and telerehabilitation through the Low Activities of Daily Living Monitoring Promag (LAMP), vs. just the standard care], showing that the telerehabilitation group slightly increased clinic visits post-intervention but slightly reduced hospital and nursing home stays. The total cost of the pre- and post-enroll days/visits was $2,767,712.90 and $2,812,250.50, respectively, while the standard care was $2,055,283.60 and $1,578,459.30 respectively.

### Telerehabilitation in cardiological disorders

Regarding the cardiological studies, five studies were selected (Table 1). These studies included telerehabilitation in chronic heart failure, cardiovascular diseases, coronary artery disease, and acute coronary syndrome or revascularization procedure. The sample size ranged from 53 to 162 patients; most were men (between 75 and 88.8%). The mean age of patients ranged from 60.5 ± 8.8 to 68.0 ± 14.0. The methodology followed in all five studies was two-arm randomized controlled trials.

Regarding the total costs per study, Hwang et al. (21) reported the costs per program performed during 12 weeks (telerehabilitation vs. center-based program) were $1,778.00 and $2,906.00, respectively, and the total health care costs per participant over 6 months showed a significant difference (*p* < 0.001) of −1,590.00 (95% CI: −2,822, −359) in favor of the telerehabilitation group (cost for telerehabilitation group = $2,325.00 vs. cost for the control group = $3,916); Maddison et al. (22) found that REMOTE-CR program cost per capita was $2,964.94 while the center-based was $5,746.08; Kraal et al. (23) reported the costs of the cardiac rehabilitation program (€336) and the home-based rehabilitation group ($314,7), being the total healthcare costs of $2,861.36 and $2,424.39, respectively; Kidholm et al. (24) included the cost of program (teledialog), rehabilitation services, and healthcare services, being the average cost per patient in the intervention group significantly higher ($5,721.73) than the control group ($4,054.02) (*p* < 0.021); Frederix et al. (25) found that the total average cost per patient was $2,160.81 in the intervention group and $2,726.06 in the control group.

Focusing on the comparison between telerehabilitation and traditional care (Table 2), some studies reported significantly lower costs in telerehabilitation, with the reductions of $1,590.00 (*p* < 0.001) (21) and $565.66 (*p* = 0.001) (25). In contrast, Kraal et al. (23) and Maddison et al. (22) found no significant differences between the treatment costs. Nevertheless, home-based training had slightly lower costs ($3,167.04 and $2,704.05) compared to the center-based training. Kidholm et al. (24) found that telerehabilitation was not cost-effective compared to traditional care, being telerehabilitation more expensive and showing no improvement in the quality of life of the patients compared to traditional care.

Finally, regarding QALY results, Frederix et al. (25) revealed an incremental cost-effectiveness ratio (ICER) of −$21,755.38/QALY, showing a reduction in the number of rehospitalization. The other studies found no differences in QALY between groups.

**FIGURE 2:**
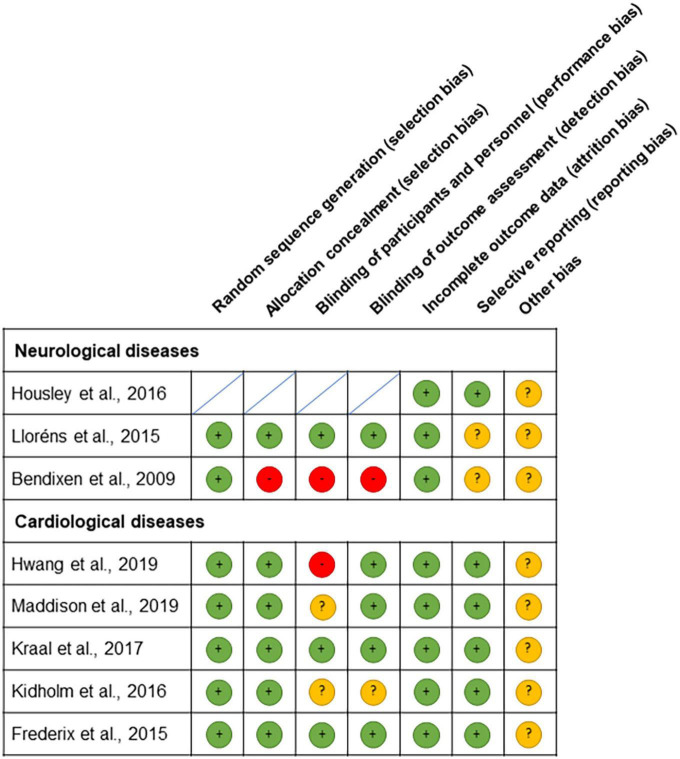
Risk of bias assessment summary according to the Cochrane’s risk of bias tool: red, green, and yellow colors indicate high, low, and unclear risk of bias, respectively.

Regarding the risk of biased assessment results, all the included studies adequately reported the random sequence generation and complete outcome data. Almost all studies reported the allocation concealment, blinding of participants, personnel and outcome assessment, and selective reporting correctly except for the following studies: Housley et al. (18) did not include an actual control group (but a simulated control group), and therefore, no random sequence, allocation concealment, or blinding could be achieved; Bendixen et al. (20) did not describe the allocation concealment and blinding; Hwang et al. (21) specified that neither subject nor treating therapist blinding could be possible in their study due to the nature of the interventions; in Maddison et al. (22), the participants could not be blinded to treatment allocation, but personnel who performed the VO_2_ max testing were blinded to treatment allocation at 12 weeks, and in the study by Kidholm et al. (24), there is not enough information about the blinding personnel and outcome assessment. The risk of bias assessment can be found in Figure 2, with red, green, and yellow colors indicating high, low, and unclear risk of bias, respectively.

In addition, focusing on the economic evaluation frame, we have also followed the CHEERS 2022 checklist. Table 3 summarizes the quality assessment of the included studies according to the CHEERS checklist. The reporting quality assessed by the CHEERS checklist varied from 74.1 to 96.3%. Among the eight publications reviewed, one study included cost analysis (18), another performed a cost-benefit analysis (19), another included cost-effectiveness (20), and the five cardiological studies conducted cost-utility analysis (21–25). Some studies did not meet a few items in the CHEERS checklist, such as the “title” specifications, the health economic analysis plan, or the discount rate.

**TABLE 3:** Quality of the included studies using CHEERS 2022 checklist.

**Item no.**	**Section/Item**	**Housley et al. (18)**	**Lloréns et al. (19)**	**Bendixen et al. (20)**	**Hwang et al. (21)**	**Maddison et al. (22)**	**Kraal et al. (23)**	**Kidholm et al. (24)**	**Frederix et al. (25)**	**Overall**
1	Title	1	0	0	1	0	1	0	0	37.5%
2	Abstract	1	1	0	1	0	1	1	1	75%
3	Background and objectives	1	1	1	1	1	1	1	1	100%
**Methods**										
4	Health economic analysis plan	0	0	0	0	0	0	0	0	0%
5	Study population	1	1	1	1	1	1	1	1	100%
6	Setting and location	1	1	1	1	1	1	1	1	100%
7	Comparators	1	1	1	1	1	1	1	1	100%
8	Perspective	1	1	1	1	1	1	1	1	100%
9	Time horizon	1	1	1	1	1	1	1	1	100%
10	Discount rate	0	0	0	1	0	0	0	0	12.5%
11	Selection of outcomes	1	1	1	1	1	1	1	1	100%
12	Measurement of outcomes	1	1	1	1	1	1	1	1	100%
13	Valuation of outcomes	1	1	1	1	1	1	1	1	100%
14	Measurement and valuation of resources and costs	1	1	1	1	1	1	1	1	100%
15	Currency, price date, and conversion	1	1	1	1	1	1	1	1	100%
16	Rationale and description of model	1	1	1	1	1	1	1	1	100%
17	Analytics and Assumptions	1	1	0	1	1	1	0	1	75%
18	Characterizing heterogeneity	N/A	N/A	N/A	N/A	N/A	N/A	N/A	N/A	
19	Characterizing distributional effects	1	1	1	1	1	1	1	1	100%
20	Characterizing uncertainty	0	1	0	1	1	1	1	1	75%
21	Approach to engagement with patients and others affected by the study	1	1	1	1	1	1	1	1	100%
**Results**										
22	Study parameters	1	1	1	1	1	1	1	1	100%
23	Summary of main results	1	1	1	1	1	1	1	1	100%
24	Effect of uncertainty	0	1	0	1	1	1	1	1	75%
25	Effect of engagement with patients and others affected by the study	1	1	1	1	1	1	1	1	100%
**Discussion**										
26	Study findings, limitations, generalizability, and current knowledge	1	1	1	1	1	1	1	1	100%
**Other relevant information**										
27	Source of funding	1	1	1	1	1	1	1	1	100%
28	Conflicts of interest	0	0	1	1	1	1	1	1	75%
	Overall quality	81.5%	85.2%	74.1%	96.3%	85.2%	92.6%	85.2%	88.9%	

1 = yes/included; 0 = no/absent; N/A = not applicable.

## Discussion

The systematic review revealed that just eight studies focused on the costs and effectiveness of neurological and cardiological telerehabilitation. According to the studies reviewed, telerehabilitation was more cost-effective than traditional rehabilitation at the clinic. Half of the studies found significant differences in cost/savings per person between the telerehabilitation performed and the traditional one at the clinic (18, 21, 22, 25). Overall, the reporting quality of the included studies varied from 74.1 to 96.3%, showing all of them the good quality. Moreover, those items that scored the lowest percentage in terms of reaching the CHEERS checklist criteria were “title,” “Health economic analysis plan,” and “discount rate.” However, most studies did not include the discount rate because the time horizon was less than 1 year. Regarding the study perspective, most of them included a healthcare system perspective.

Although there are several reviews regarding telerehabilitation in neurological (i.e., patients with stroke) (26, 27) and cardiological diseases (i.e., heart failure) (28), just a few studies met our inclusion and exclusion criteria. Specifically, few studies were focused on telerehabilitation and included cost evaluations. This is the first systematic review focused on the economic evaluation of telerehabilitation in neurological and cardiological diseases. However, it is highlighted that in order to make proper clinical decisions and decide whether a new telerehabilitation program is good enough, from the clinical and economic perspective, to be implemented in the public or private health system, cost needs to be considered, and therefore, cost-effectiveness analyses are needed.

### Neurological diseases

As part of the vCare project, the systematic review focused on neurological diseases in general and stroke and Parkinson’s disease in particular. However, only three studies included costs and clinical results of telerehabilitation in patients after stroke. None of these studies included QALY assessment, but cost, feedback questionnaires, clinical visits, and home stays. A study by Housley et al. (18) was the only neurological study that found significant differences in telerehabilitation cost/saving per person compared to traditional rehabilitation at the clinic. Lloréns et al. (19) also found lower costs of the telerehabilitation program, but they did not specify whether the differences were statistically significant. Bendixen et al. (20) examined healthcare costs after 12 months from the telerehabilitation (LAMP) intervention and found no significant cost differences. The telerehabilitation group made more clinic visits while decreasing hospital and nursing home stays.

Regarding clinical results, patients gave positive feedback on the telerehabilitation performed after stroke and showed significant improvements in balance and gait. In contrast, Lloréns et al. (19) did not find significant differences in the feedback questionnaires, usability, or motivation between the telerehabilitation group and the traditional rehabilitation at the clinic.

### Cardiological diseases

Telerehabilitation in cardiological diseases is also relevant since cardiac rehabilitation is an essential component of improving physical, psychological, and social functioning (28, 29), but few studies have focused on assessing the economic evaluation or the cost-effectiveness differences between telerehabilitation and traditional rehabilitation. After an exhaustive literature revision, we found five studies that met our criteria and included costs and clinical outcomes in cardiological diseases such as heart failure or acute coronary syndrome. These studies analyzed rehabilitation cost-utility, including QALY measured with the EQ-5D or SF-36. In total, three cardiological rehabilitation studies found significant differences in cost/savings per person (21, 22, 25), although most of the studies did not find statistically significant differences in QALY between groups of rehabilitation. Specifically, one of the cost-effectiveness studies found QALY significant differences between groups, the intervention group more effective (25). However, this study’s intervention group differed from other studies reviewed since the experimental group performed a telerehabilitation program in addition to center-based cardiac rehabilitation. In contrast, the control group just performed the center-based rehabilitation itself (25). In addition, Frederix et al. (30) performed a 2-year follow-up study finding clinically significant differences between the telerehabilitation group and the center-based rehabilitation. Generally, the telerehabilitation group significantly increased their physical activities, perceived health-related quality of life, and the QALY at follow-up (30). However, these authors found that telerehabilitation added to the standard center-rehabilitation was more effective and costly than the standard center-rehabilitation alone. On the other hand, Hwang et al. (21) and Kidholm et al. (24) revealed non-significant differences in QALY between groups, concluding that telerehabilitation might be as effective as traditional rehabilitation. Furthermore, Hwang et al. (21) found that telerehabilitation was significantly less costly. Maddison et al. (22) also found no difference in QALY, even if medication costs were lower in the telerehabilitation group. However, adverse events were higher during treatment in the telerehabilitation group (22). Kraal et al. (23) showed similar QALY between groups, but almost all components were lower in the telerehabilitation group. They also found similar treatment adherence and clinical improvements in both groups, but patients had higher satisfaction in the telerehabilitation group (23).

### Duration of the telerehabilitation

The telerehabilitation time ranged from 6 to 48 weeks in the studies reviewed (neurological and cardiological diseases). This difference could be a limitation in making a comparison between them. However, most of the cardiological studies reviewed performed telerehabilitation for 12 weeks.

### Strengths and limitations

There is an increased interest in healthcare spending in Europe and worldwide, specifically in telerehabilitation and care for elders. Chronic illness contributes to disability, diminishes the quality of life, and decreases health and long-term care costs (31). Traditional care and rehabilitation imply inpatient care, skilled multidisciplinary clinicians, outpatient clinics, and/or home health visits. As life expectancy is increasing, the availability of cost-effective telerehabilitation programs is crucial for neurological, cardiological, or chronic diseases and active aging. Moreover, telerehabilitation could expand the access to perform rehabilitation for people that could not have access to traditional clinic care either for personal, geographical, economic reasons or due to the public health system (18, 32). Moreover, in the COVID-19 pandemic, telerehabilitation has emerged as a valuable tool for the continuity of care at home, offering professionals a rapid learning experience in implementing telerehabilitation with their patients in a satisfactory manner (33, 34).

Our systematic review showed that comparing costs and the cost-effectiveness of different interventions is crucial for making evidence-based decisions regarding telerehabilitation implementation in health systems. Telerehabilitation seems to be as effective as traditional rehabilitation and even less costly. Patients who performed telerehabilitation presented greater satisfaction and adherence to treatment and rehabilitation. However, few studies reported economic evaluation of the rehabilitation performed, and in those that included it, costs varied across different intervention designs. Future clinical trials should include cost-effectiveness analysis as a relevant measure to decide whether telerehabilitation is a good option to be implemented in public health systems. In addition, future research should also consider comparing different telerehabilitation and interventions to determine which are the ones that best meet the needs of each disease.

The studies reviewed found several limitations that also should be taken into account. First, the small number of studies that met the inclusion criteria for this systematic review limits the generalization of the study’s conclusions. Moreover, the study design (i.e., not including a control group) and the lack of blinding during the patient allocation process restrict the effectiveness measurement. Finally, the duration of the treatment is variable among studies, and the sample (i.e., small sample size, limited age range, or an unbalanced number of men and women) produces variability in the findings. Future studies should overcome these limitations to obtain more consistent and generalizable findings.

### Conclusion

In conclusion, telerehabilitation is a suitable alternative to traditional rehabilitation care in post-stroke patients and in cardiological diseases, especially in remote or underserved areas. More extensive economic evaluation studies are needed to evaluate the cost-effectiveness and the health-related quality of life of patients who perform telerehabilitation.

## Data availability statement

The raw data supporting the conclusions of this article will be made available by the authors, without undue reservation.

## Author contributions

RD and MD-C contributed equally to the conception, design of the study, and interpretation of the results. RD, MD-C, IU-A, and SG-L contributed to the acquisition and analysis of data. RD, MD-C, and IU-A contributed to initial and final manuscript. All authors contributed to the critical revision of the manuscript and final version approval.
