# Supplementary material for: Costs and effects of telerehabilitation in neurological and cardiological diseases: A systematic review
Source: Front Med (Lausanne). 2022 Nov 29;9:832229. doi: 10.3389/fmed.2022.832229 (PMC9745081; doi:10.3389/fmed.2022.832229)
Supplement: Supplementary file 1 [file Table_1.DOCX]

**Supplementary Table 1: keywords organized by topics.**

| **Keywords related to cost** | **Keywords related to telerehabilitation** | **Keywords related to cardiological rehabilitation** | **Keywords related to neurological rehabilitation** |
| --- | --- | --- | --- |
| Costs and cost analysis  Cost  Cost-benefit analysis  Cost-utility  Cost effectiveness  Hospital costs  Cost control  Cost utility analysis  Cost minimization analysis  Cost effectiveness analysis | Telerehabilitation  Virtual rehabilitation  Virtual reality  User-computer interface  Clinical competence  Computer simulation  Computer-assisted instruction  Virtual training  Virtual rehabilitation system | Cardiac rehabilitation  Heart rehabilitation  Cardiac rehabilitation  Heart rehabilitation  Heart failure | Stroke rehabilitation  Parkinson rehabilitation  Neurorehabilitation  Neurological rehabilitation |
