# Supplementary material for: Costs and effects of telerehabilitation in neurological and cardiological diseases: A systematic review
Source: Front Med (Lausanne). 2022 Nov 29;9:832229. doi: 10.3389/fmed.2022.832229 (PMC9745081; doi:10.3389/fmed.2022.832229)
Supplement: Supplementary file 2 [file Table_2.DOCX]

Supplementary Table 2. Keywords strategy.

|  | **Database** | **Number of results** |
| --- | --- | --- |
|  | **MEDLINE** | |
| 1 | “COSTS AND COST ANALYSIS” [Mesh] OR “COST” [mp]) AND (“Rehabilitation Research” [Mesh] OR “Physical and Rehabilitation Medicine” [Mesh] OR “Neurological Rehabilitation” [Mesh] OR “Stroke Rehabilitation” [Mesh] OR “Hospitals, Rehabilitation” [Mesh] OR “Cardiac Rehabilitation” [Mesh] OR “Rehabilitation” [mp] | 33 |
| 2 | Cost-Benefit Analysis [Mesh] OR cost-utility [mp]) AND (“Rehabilitation Research” [Mesh] OR “Physical and Rehabilitation Medicine” [Mesh] OR “Neurological Rehabilitation” [Mesh] OR “Stroke Rehabilitation” [Mesh] OR “Hospitals, Rehabilitation” [Mesh] OR “Cardiac Rehabilitation” [Mesh] OR “Rehabilitation” [mp]) AND (“Telerehabilitation” [Mesh] OR “virtual rehabilitation” [mp] | 23 |
| 3 | Cost-Benefit Analysis [Mesh] OR cost-utility [mp]) AND (Virtual Reality [Mesh] OR User-Computer Interface [Mesh] OR Clinical Competence [Mesh] OR Computer Simulation [Mesh] OR Computer-Assisted Instruction [Mesh] OR VIRTUAL TRAINING [mp] | 22 |
| 4 | “Cost-Benefit Analysis” [Mesh] OR “Cost effectiveness” [mp]) AND (“Rehabilitation” [Mesh] OR “Rehabilitation [mp]) AND “Telerehabilitation” [Mesh] OR “Telerehabilitation” [mp] | 71 |
| 5 | “Cardiac Rehabilitation” [Mesh] OR “Heart Failure” [Mesh] OR “Exercise Therapy” [Mesh] OR “heart rehabilitation” [mp]) AND (“Telerehabilitation” [Mesh] OR “Telerehabilitation” [mp]) AND (“Cost-Benefit Analysis” [Mesh] OR “Cost effectiveness” [mp] | 20 |
| 6 | “Neurological rehabilitation” [Mesh] OR “neurorehabilitation” [mp]) AND (“Telerehabilitation” [Mesh] OR “Telerehabilitation” [mp]) AND (“Cost-Benefit Analysis” [Mesh] OR “Cost benefit analysis” [mp] | 1 |
| 7 | “rehabilitation research” [Mesh] OR “rehabilitation research” [mp]) AND (“Hospital costs” [Mesh] OR “Hospital costs” [mp] OR “cost control”[Mesh] OR “cost control [mp] OR “cost-benefit analysis”[Mesh] OR cost utility analysis [mp] | 7 |
|  | **EMBASE** | |
| 1 | “Cost benefit analysis” [emtree] OR “cost minimization analysis” [emtree] OR “cost” [emtree] OR “cost effectiveness analysis” [emtree] OR “cost utility analysis”/ or COST.mp) AND (“heart rehabilitation” [emtree] OR “rehabilitation” [emtree] OR “stroke rehabilitation” [emtree] OR “rehabilitation medicine” [emtree] OR “rehabilitation research” [emtree] OR rehabilitation mp) AND (telerehabilitation [emtree] OR VIRTUAL REHABILITATION [mp] | 181 |
| 2 | “Cost benefit analysis” [emtree] OR “cost minimization analysis” [emtree] OR “cost” [emtree] OR “cost effectiveness analysis” [emtree] OR “cost utility analysis”/ or COST.mp) AND (“neurorehabilitation” [emtree] OR “neurological rehabilitation”mp) AND (telerehabilitation [emtree] OR VIRTUAL REHABILITATION [mp] | 11 |
| 3 | “Cost benefit analysis” [emtree] OR “cost minimization analysis” [emtree] OR “cost” [emtree] OR “cost effectiveness analysis” [emtree] OR “cost utility analysis”/ or COST.mp) AND (“heart rehabilitation” [emtree] OR cardiac rehabilitation mp.) AND (telerehabilitation [emtree] OR VIRTUAL REHABILITATION [mp] | 25 |
| 4 | “cost utility analysis” [emtree] OR “cost benefit analysis” [emtree] OR “cost minimization analysis” [emtree] OR “cost effectiveness analysis” [emtree] OR cost.mp) AND (“heart rehabilitation” [emtree] OR. “rehabilitation” [emtree] OR “stroke rehabilitation” [emtree] OR “rehabilitation research” [emtree] OR “rehabilitation medicine” [emtree] OR “pulmonary rehabilitation” [emtree] OR r“virtual rehabilitation system”[emtree] OR REHABILITATION. [mp] | 35 |
|  | Other sources | 2 |
